# Association between childhood maltreatment and cortical folding in women with eating disorders

**DOI:** 10.1192/j.eurpsy.2024.641

**Published:** 2024-08-27

**Authors:** R. Cerra, N. Attianese, M. Battipaglia, R. Ceres, S. Donato, A. M. Monteleone, P. Monteleone, G. Cascino

**Affiliations:** ^1^Department of Mental, Physical Health and Preventive Medicine, University of Campania “Luigi Vanvitelli”, Naples; ^2^Department of Medicine, Surgery and Dentistry, University of Salerno “Scuola Medica Salernitana”, Salerno, Italy

## Abstract

**Introduction:**

Childhood maltreatment (CM) is associated with distinct clinical and biologi- cal characteristics in people with eating disorders (EDs). The measurement of local gyrification index (lGI) may help to better characterize the impact of CM on cortical structure.

**Objectives:**

The objective of this study was to investigate the association of CM with lGI in women with EDs.

**Methods:**

Twenty-six women with anorexia nervosa (AN) and 24 with bulimia nervosa (BN) underwent a 3T MRI scan. All partici- pants filled in the Childhood Trauma Questionnaire. All neuroimaging data were processed by FreeSurfer. LGI maps underwent a general linear model to evaluate differences between groups with or without CM. People with AN and BN were merged together.

**Results:**

Based on the Childhood Trauma Questionnaire cut- off scores, 24 participants were identified as maltreated and 26 as non- maltreated. Maltreated people with EDs showed a significantly lower lGI in the left middle temporal gyrus compared with non-maltreated people, whereas no differences emerged in the right hemisphere between groups.

**Conclusions:**

The present study showed that in people with EDs, CM is associated with reduced cortical folding in the left middle temporal gyrus, an area that could be involved in ED psychopathology. This finding corroborates the hypothesis of a ‘maltreated ecophenotype’, which argues that CM may allow to biologically, other than clinically, distinguish individuals with the same psychiatric disorder.

**Disclosure of Interest:**

None Declared

